# Multiprotein GLI Transcriptional Complexes as Therapeutic Targets in Cancer

**DOI:** 10.3390/life12121967

**Published:** 2022-11-24

**Authors:** Fan Yang, Daniel T. Wynn, Chen Shen, Nagi G. Ayad, David J. Robbins

**Affiliations:** 1 Miller School of Medicine, University of Miami, Miami, FL 33136, USA; 2 Department of Oncology, Lombardi Comprehensive Cancer Center, Georgetown University, Washington, DC 20057, USA

**Keywords:** GLI, transcriptional complex, hedgehog signaling, epigenetic regulation

## Abstract

The Hedgehog signaling pathway functions in both embryonic development and adult tissue homeostasis. Importantly, its aberrant activation is also implicated in the progression of multiple types of cancer, including basal cell carcinoma and medulloblastoma. GLI transcription factors function as the ultimate effectors of the Hedgehog signaling pathway. Their activity is regulated by this signaling cascade via their mRNA expression, protein stability, subcellular localization, and ultimately their transcriptional activity. Further, GLI proteins are also regulated by a variety of non-canonical mechanisms in addition to the canonical Hedgehog pathway. Recently, with an increased understanding of epigenetic gene regulation, novel transcriptional regulators have been identified that interact with GLI proteins in multi-protein complexes to regulate GLI transcriptional activity. Such complexes have added another layer of complexity to the regulation of GLI proteins. Here, we summarize recent work on the regulation of GLI transcriptional activity by these novel protein complexes and describe their relevance to cancer, as such GLI regulators represent alternative and innovative druggable targets in GLI-dependent cancers.

## 1. The Hedgehog Signaling and GLI Regulation

The Hedgehog (HH) signaling pathway orchestrates both embryonic development and tissue homeostasis in adults (reviewed in [1,2]). Importantly, altered HH signaling as well as the ectopic activation of its effectors, GLI transcription factors, are implicated in multiple types of cancers, most notably in basal cell carcinoma (BCC), medulloblastoma (MB), and rhabdomyosarcoma (reviewed in [1]). This has driven great interest in developing clinically relevant inhibitors of HH signaling. Among such inhibitors, those targeting Smoothened (SMO), a rate-limiting activator of the HH signaling pathway [3], have had the most success. Currently, three small-molecule inhibitors targeting SMO are FDA-approved: vismodegib and sonidegib for BCC, and glasdegib for acute myeloid leukemia [4,5,6]. However, mutations at or downstream of SMO often render these cancers resistant to SMO inhibitors [7,8]. As GLI proteins are the ultimate effectors of the HH signaling pathway and are themselves capable of driving oncogenesis, they are attractive targets for drugs that could circumvent SMO inhibitor resistance [9]. Unfortunately, transcription factors (TFs) have been notoriously difficult to drug (reviewed in [10]). Further, though multiple small molecule inhibitors that directly target GLI proteins have been produced [11,12,13,14,15,16], their poor pharmacokinetics and toxicity have limited their clinical development [17,18,19,20,21,22]. As such, an alternative indirect strategy for drugging GLI proteins has emerged in which a regulator within a multi-protein GLI TF complex could instead be targeted. The focus of this review is to describe such multiprotein GLI transcriptional complexes and to provide examples of their druggability.

**Figure 1:**
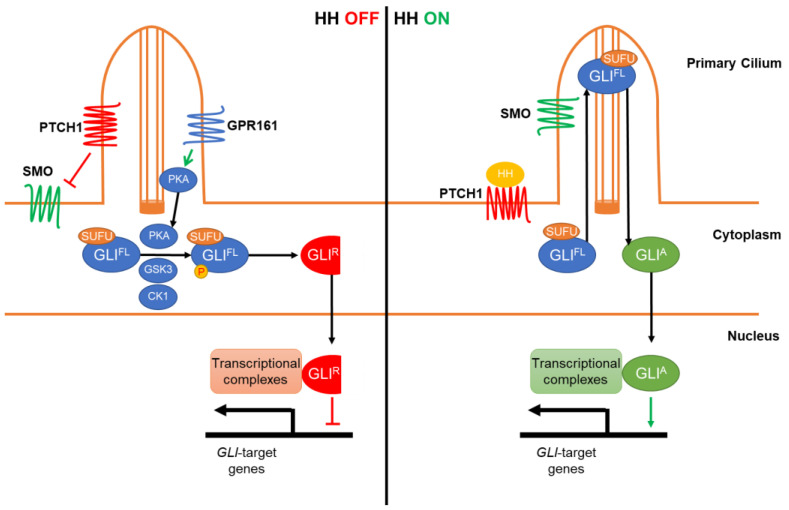
The canonical Hedgehog signaling pathway. In the absence of Hedgehog (HH) ligands (HH OFF), Patched 1 (PTCH1) localizes at primary cilia and inhibits Smoothened (SMO). G-protein-coupled receptor 161 (GPR161) also localizes at primary cilia and activates Protein kinase A (PKA). Full-length GLIs (GLI^FL^) are thus phosphorylated by the activated PKA, and then by Glycogen synthase kinase-3 (GSK3) and Casein kinase 1 (CK1). The phosphorylated GLI^FL^ are subsequently proteolysed into c-terminal truncation repressor forms (GLI^R^) that function as transcriptional repressors of their target genes in cooperation with co-factors. In the presence of HH ligands (HH ON), HH interacts with and inactivates PTCH1, releasing its inhibition on SMO. Consequently, GLI^FL^ and Suppressor of fused (SUFU) accumulate at the tips of primary cilia. Activated SMO also accumulates at primary cilia, where it induces the dissociation of GLI^FL^ from SUFU and the transformation of GLI^FL^ into the activator forms (GLI^A^). GLI^A^ are then transported into the nucleus where they form complexes with other co-factors and function as transcriptional activators to initiate the expression of their target genes.

The canonical HH signaling pathway refers to GLI activation through a HH-Patched 1 (PTCH1)-SMO axis (Figure 1). In the absence of HH ligands (Sonic, Indian, or Desert Hedgehog), the 12-transmembrane receptor PTCH1 localizes on the surface of primary cilia, organelles projecting from the surface of most vertebrate cells [23,24]. PTCH1 functions as a negative regulator of the HH signaling pathway by inhibiting the activity of SMO, a seven-transmembrane protein that plays a rate-limiting role in activating the pathway [3,25,26]. The mechanism of SMO inhibition by PTCH1 is likely mediated by cholesterol, an agonist of SMO, whose accessibility to SMO in the primary cilia is restricted by active PTCH1 [27]. Furthermore, another negative regulator of the signaling pathway, G-protein-coupled receptor 161 (GPR161), localizes at primary cilia and activates Protein kinase A (PKA) [28]. PKA and other kinases subsequently phosphorylate GLI proteins, resulting in their proteolytic processing into transcriptional repressors [29,30,31,32]. In the presence of HH, HH proteins bind to their major receptor PTCH1 along with various co-receptors [33,34,35,36]. HH binding inactivates PTCH1, promoting its removal from primary cilia [2,27,37]. SMO subsequently accumulates in the primary cilia, where it induces the removal of GPR161 from cilia [28,38], and activates GLI proteins [39,40,41]. Upon SMO activation, GLI proteins are converted into transcriptional activators and translocate to the nucleus where they initiate the expression of their target genes [2,37]. The mechanism of GLI activation by SMO is likely through the release of GLIs from Suppressor of fused (SUFU) in primary cilia, which normally functions to sequester GLI proteins in the cytoplasm [42,43,44].

There are three GLI proteins: GLI1, 2, and 3 [45]. Among these, GLI1 functions exclusively as a transcriptional activator [46], while GLI2 and GLI3 harbor both activator and repressor domains [29,30]. In the absence of HH, a phosphorylation-mediated C-terminal truncation transforms full-length GLI2 and GLI3 into transcriptional repressors. However, in the presence of HH, full-length GLI2 and GLI3 are converted into transcriptional activators [29,30,31,32]. GLI2 is the primary transcriptional activator whereas GLI3 functions primarily as a transcriptional repressor [47,48,49]. In general, the activity of GLI proteins is regulated in four ways, via regulation of their (i) mRNA expression, (ii) subcellular localization, (iii) stability (reviewed in [2,37]), and (iv) transcriptional activity. The ability of GLI transcription factors to control target gene expression is predominantly regulated by the transcriptional co-factors of GLI (see below).

In addition to canonical HH signaling, GLI activity is also regulated via mechanisms independent of the SHH-PTCH1-SMO axis [50]. Such non-canonical GLI regulation is often implicated in cancer and involves all four forms of GLI regulation described above. For example, GLI2 expression is ectopically induced by the TGF-β/SMAD signaling in melanoma and breast cancer [51]. It has also been observed that GLI stability and nuclear localization is enhanced by Phosphoinositide 3-kinase (PI3K)/AKT serine/threonine kinase (AKT) signaling in leukemia, esophageal adenocarcinoma, pancreatic cancer, melanoma, and MB [52,53,54,55,56,57]. Furthermore, GLI transcriptional activity is upregulated by Serum response factor (SRF) and Megakaryoblastic leukemia 1 (MKL1) in SMO inhibitor resistant BCC ([58] and will be discussed below).

The transcription of GLI-target genes is achieved by cooperation between GLI proteins and other transcriptional co-factors, including general transcription machinery, epigenetic regulators, and other transcription factors (Table 1). Many of these transcriptional co-factors function within multi-protein complexes that may serve as alternative targets for GLI inhibition. In the sections below, we will review the known multi-protein GLI transcriptional complexes, describe their roles in GLI-dependent cancers, and discuss their potential as therapeutic targets in these cancers.

## 2. Multi-Protein GLI Transcriptional Complexes

### 2.1. General Transcription Machinery

**Figure 2:**
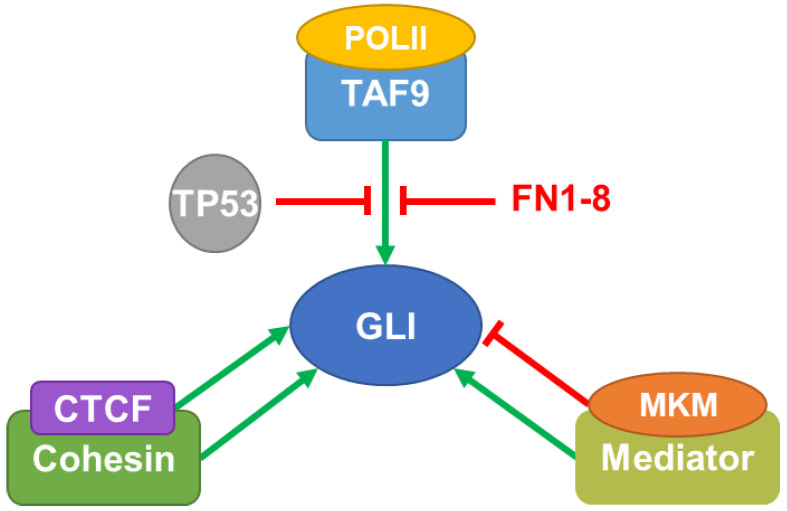
GLI proteins form transcriptional complexes with the general transcription machinery. GLI proteins interact with TBP Associated Factor 9 (TAF9) to initiate the recruitment of RNA polymerase II (POLII) and the subsequent target gene transcription. The interaction between TAF9 and GLI 1/2 can be interrupted by TP53 or a small molecule inhibitor, FN1-8. GLI3 associates with Mediator and Mediator kinase module (MKM). It is likely that Mediator enhances GLI transcriptional activity while the MKM inhibits it. The Cohesin-CCCTC-binding factor (CTCF) complex co-occupies GLI-target genes along with GLI proteins to enhance their transcription.

Multiple components of the general transcription machinery associate with GLI proteins to regulate their transcriptional activity, including the preinitiation complexes, Mediator, and Cohesin (Figure 2). Though the inhibition of this general transcription machinery is often considered toxic, an alternative strategy of interrupting their interaction with specific TFs provides the potential for selective inhibition. Thus, defining the interactions between GLI and general transcription machinery provides a better understanding of GLI transcriptional regulation as well as potential druggable targets. In this section, we will cover the current knowledge on this area.

#### 2.1.1. Transcription Factor II D Complex and TP53

The recruitment of RNA polymerase II (POLII) requires the stepwise assembly of multiple-protein preinitiation complexes, which generally starts with the recruitment of Transcription factor II D (TFIID) by specific transcription factors (reviewed in [110]). TFIID is comprised of 13 TBP Associated Factors (TAFs) [111]. GLI1 and GLI2 interact with TAF9 within this complex at the ‘FXXΦΦ’ (F: phenylalanine, X: any amino acid, and Φ: any hydrophobic amino acid) domain in their transcription activation region, which is a conserved motif also found in herpes simplex viral protein 16 and TP53 [60]. TAF9-GLI1/2 interaction was confirmed in cell-free assays and cancer cells, where TAF9 functions as a coactivator to enhance GLI transcriptional activity [59,60]. This interaction can in turn be interrupted by TP53, which can indirectly repress GLI1 activity by competing with GLI1 for TAF9 binding [60]. Notably, TP53 inhibits the activity of GLI on multiple levels [112,113] and, consistent with such a role, its mutation is associated with poor patient survival in SHH subgroup MB (SHH-MB) [114,115], a subgroup of MB characterized by hyperactivation of HH/GLI signaling [116]. Importantly, small molecules that mimic the ‘FXXΦΦ’ motif of GLI proteins, such as FN1-8 (Table 2), are capable of interrupting the interaction between TAF9 and GLIs without affecting the interaction between TAF9 and TP53, suggesting that selective inhibition of GLI complexes is feasible [59]. These inhibitors significantly suppress the growth of GLI-dependent lung cancer cells ex vivo and in vivo by inhibiting GLI transcriptional activity [59,117].

#### 2.1.2. Mediator Complex

Mediator is a large protein complex comprised of 26 subunits that is reversibly associated with the functionally independent four-subunit Mediator kinase module (MKM) [120]. MKM is composed of Cyclin dependent kinase 8 (CDK8), mediator complex subunit 12 (MED12), MED13, and Cyclin C [120]. Mediator interacts with transcription factors at enhancer regions of chromatin and with preinitiation complexes components at core promoter regions to promote chromatin looping [121]. GLI3 interacts with both the Mediator (MED1, MED14, MED6, and MED23) and the MKM (MED12 and CDK8) [61]. Interestingly, Mediator and MKM appear to play different roles in GLI signaling. MKM inhibits GLI activity in a manner dependent on CDK8 kinase activity [61,62]. Nonetheless, a CRISPR-based screen identified the MKM component MED12, but not CDK8, as well as Mediator components (MED1, 7, 19, 25, 26, 27, 30, and 31) as positive regulators of GLI signaling [63]. Given that CDK8 can directly phosphorylate TFs to either activate or inhibit their function [120], CDK8 is likely capable of phosphorylating GLI to modulate its activity independently of the Mediator main body.

#### 2.1.3. Cohesin-CCCTC-Binding Factor Complex

Aside from its well-known function in chromosome segregation, Cohesin can also cooperate with CCCTC-binding factor (CTCF) to enhance gene transcription by forming chromatin loops that enable long-range communication between enhancers and promoters [122,123]. In GLI-mediated transcription, the Cohesin-CTCF complex functions as a GLI co-activator. Specifically, RAD21 Cohesin complex component, a Cohesin structural subunit, and Nipped-B, a protein that loads cohesin complex onto DNA [123], were identified as positive regulators of HH signaling [64]. Further, consistent with the Cohesin-CTCF complex regulating GLI-mediated transcription, GLI1- and GLI2 binding regions within the human genome largely overlap with CTCF-binding regions [65].

### 2.2. Epigenetic Regulators

Epigenetic regulation refers to the modifications of histones or DNA that regulate chromatin structure and gene expression [124]. These modifications are in turn “read”, “written”, or “erased” by epigenetic regulators [124], a number of which associate with GLI proteins to regulate GLI-mediated transcription. Importantly, many epigenetic regulators are druggable [125], providing alternative targets to inhibiting GLI proteins directly.

#### 2.2.1. Bromodomain-Containing 4 Complexes and SRY-Box Transcription Factor 2

Bromodomain-containing 4 (BRD4) is a well-established epigenetic activator that recognizes histone acetylation, a hallmark of active gene transcription [126]. It associates with promoters or enhancers and subsequently recruits the positive transcription elongation factor complex (P-TEFb) and POLII to promote gene transcription [126]. BRD4 interacts with GLI1 and GLI2 and colocalizes with them on the promoter regions of GLI-target genes to induce RNA expression [66,67]. BRD4 also interacts with SRY-box transcription factor 2 (SOX2), another GLI transcription co-activator [89,127], to co-transactivate the *GLI1* promoter to activate its transcription [68]. SOX2-mediated GLI activation is best characterized in SHH-MB, where a SOX2-enriched cancer cell cluster harbors noncanonical GLI activation to render them resistant to SMO inhibition [71,90,91]. The BRD4-SOX2-GLI1 transcriptional complex has also been seen in melanoma, where it acts to transactivate GLI-target genes such as *GLI1* [68] and *ST3GAL1* [88]. Importantly, multiple BRD4 inhibitors including JQ1, I-BET151, CPI203, BMS-986158, MZ1, and ZEN-3365, have been shown to inhibit GLI-mediated transcription and the growth of GLI-dependent cancers ([66,67,68,69,70,71,118,128] and Table 2).

#### 2.2.2. Histone/GLI-Dual Regulators

**Figure 3:**
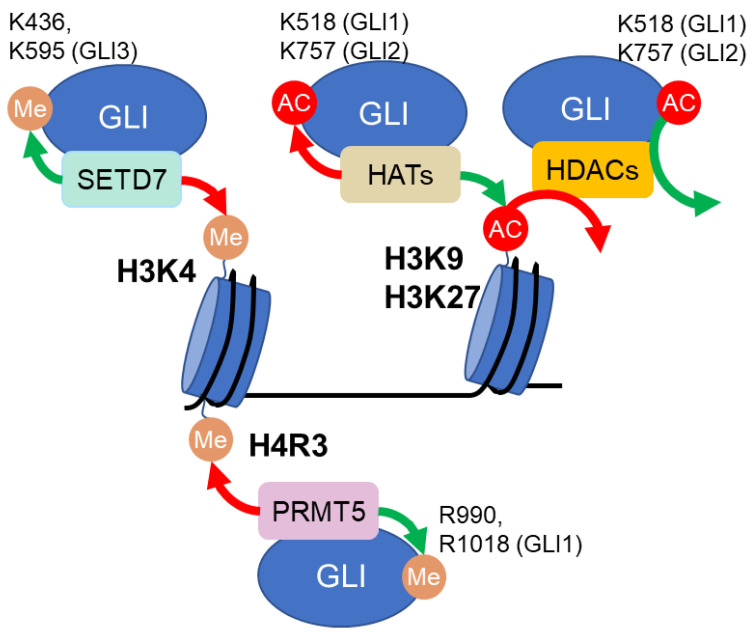
Several epigenetic regulators can modify both histones and GLI proteins, with opposing effects on GLI activity. HATs, HDACs, SETD7, and PRMT5, can modulate the posttranslational modifications of both histones and GLI proteins (targeted residues are labeled), with opposing effects on GLI signaling. Green and red arrows indicate actions that potentiate and repress GLI signaling, respectively.

Histone acetylases (HATs), histone deacetylases (HDACs), Protein Arginine Methyltransferase 5 (PRMT5), and SET Domain Containing 7 (SETD7) are categorized together in this section because they modify both histones and GLI proteins with opposing effects on GLI activity (Figure 3). Specifically, histone acetylation, which is generally linked with transcriptional activation, is catalyzed by HATs and reversed by HDACs [129]. When interacting with GLI proteins in transcriptional complexes, HAT-mediated histone acetylation activates GLI-mediated transcription (Figure 3). Specifically, the HAT CREB binding protein (CREBBP, also known as CBP or KAT3A) interacts with GLI3 to co-activate the transcription of *GLI1* [72]. Another HAT, P300/CBP-associated factor (PCAF, also known as KAT2B), associates with GLI1 and induces H3K9 acetylation on the promoters of GLI-target genes to activate GLI target genes [73]. In contrast, HDAC-mediated histone deacetylation inhibits GLI-mediated transcription (Figure 3). The HDAC1-SKI proto-oncogene (SKI) complex interacts with the repressor forms of GLI2 and GLI3 to mediate GLI transcriptional repression [74]. Consistent with its role as a transcriptional inhibitor, HDAC1 was found to occupy GLI-binding regions and remove H3K27 acetylation at HH-responsive enhancers [75]. In addition to HDAC1, HDAC3 can also remove the acetylation of H3K27 at the *GLI1* promoter to inhibit BRD4 recruitment and the subsequent expression of *GLI1* [76]. Two repressive histone methyltransferases, PRMT5 and SETD7, can also epigenetically inhibit GLI-mediated transcription (Figure 3). Specifically, PRMT5 complexes with MENIN1 (MEN1) to occupy the promoters of two GLI-target genes, *GLI1* and *GAS1* [77,78]. PRMT5 then symmetrically methylates H4R3 to epigenetically inhibit *GLI1* and *GAS1* expression and, as a result, attenuates GLI activity [77,78]. Similarly, SETD7 regulates the proliferation, invasion, and metastasis of breast cancer by inhibiting *GLI1* transcription [79].

Interestingly, HATs, HDACs, PRMT5, and SET7 can also modify GLI activity directly via directly posttranslational modifying GLI proteins (Figure 3). Though not the focus of this review, these direct GLI modifications are often dysregulated in cancer and thus included here. Briefly, GLI1 and GLI2 can be acetylated by CBP/E1A binding protein p300 (EP300, also known as KAT3B) [130,131,132] or PCAF [132] and consequently exhibit inhibited transcriptional activity [130,131]. This GLI acetylation can be reversed by HDAC1, 2, 6, and Sirtuin 1 (SIRT1) [130,131,133,134,135]. Further, PRMT5 and SETD7 can directly methylate GLI1 and GLI3, respectively, to increase their stability or DNA binding affinity in a manner that enhances HH signaling activity [136,137].

#### 2.2.3. Polycomb Repressive Complex 2

Polycomb repressive complex 2 (PRC2) is an epigenetic inhibitor, functioning through mono-, di- and tri-methylating H3K27 [138]. It is likely that PRC2 cooperates with the repressor forms of GLI to decrease the expression of a subset of GLI-target genes. Two core subunits of PRC2, Suppressor of Zeste 12 Homolog (SUZ12) and Enhancer of Zeste Homolog 2 (EZH2), interact with GLI2 and 3 [80]. Further, in the absence of HH, PRC2-mediated H3K27me3 represses the expression of a subset of GLI target genes, and such repression is SUZ12 or EZH2 dependent [81]. However, these finding remain controversial as it was also suggested that PRC2 may not be the primary repressive mechanism for most GLI-target genes because, in the absence of HH ligands, most HH-responsive GLI binding loci lack enrichment of the PRC2 biomarker H3K27me3 [75,139].

#### 2.2.4. Switch/Sucrose Non-Fermentable Complex

Switch/sucrose non-fermentable (SWI/SNF) complexes function to enhance the transcription of specific genes by sliding nucleosomes along genomic DNA or ejecting nucleosomes from DNA to expose that region of DNA [140]. SWI/SNF complexes exist in multiple subfamilies that share a set of core subunits: either of the ATPases SMARCA4 (also known as BRG1) or SMARCA2 (also known as BRM), which couple ATP hydrolysis with chromatin remodeling, and SMARCC1 (also known as SRG3), SMARCC2 (also known as BAF170), and SMARCD1, which target and regulate the remodeling activity [140,141]. These core subunits have been shown to occupy the regulatory regions of GLI-target genes and physically interact with GLI proteins: SMARCC1 with GLI2, GLI3, and the repressor form of GLI3 [80]; SMARCC2 with GLI1 [82]; SMARCA4 with GLI1, 2, 3, and the repressor form of GLI3 [83]; and SMARCA2 with GLI1 [84]. SWI/SNF complexes play a dual role in GLI-mediated transcription, as repressors during basal level GLI signaling and as activators when GLI is activated. Specifically, SMARCA4 represses the basal expression of GLI-target genes and is required to fully activate those genes in response to HH stimulation [83,85]. Interestingly, SMARCA4 interacts with HDAC1 and 2, suggesting that it cooperates with HDACs to modulate GLI transcriptional activity [83]. In GLI-hyperactivated rhabdomyosarcoma [142], SMARCA2 cooperates with GLI1 to enhance the chromatin accessibility of GLI-target genes, increasing their expression [84]. SMARCC1 differentially regulates the H3K27me3 level of GLI-target genes, potentially through PRC2, to activate or repress their expression in a manner dependent on the level of GLI activation [80]. An additional regulatory subunit of the SWI/SNF complexes, SMARCB1 (also known as SNF5), also interacts with GLI1 and colocalizes on GLI-target genes to represses their basal expression [82].

#### 2.2.5. Ubiquitin Like with PHD and Ring Finger Domains 1-DNA Methyltransferase 1 Complex

A Ubiquitin like with PHD and ring finger domains 1 (UHRF1)-DNA methyltransferase 1 (DNMT1)-proliferating cell nuclear antigen (PCNA) complex functions in DNA methylation inheritance, copying the CpG methylation pattern from the template strand into the newly synthesized DNA strand during replication [143]. Importantly, UHRF1 and DNMT1 can also function as transcriptional co-repressors by associating with specific transcription factors to mediate target gene methylation and subsequent gene silencing [144,145,146]. Consistent with this latter function, we recently described a UHRF1/DNMT1/GLI complex that lacks PCNA [86]. However, rather than inhibiting GLI signaling, this complex potentiates GLI activity and the growth of SHH-MB [86]. An FDA-approved DNMT1 inhibitor, 5-Azacytidine, effectively disrupts this protein complex and attenuates SHH-MB growth ex vivo and in vivo ([86] and Table 2). Mechanistically, we hypothesize that UHRF1 and DNMT1 might inhibit the expression of a selective subset of GLI-target genes that function as repressors of GLI signaling [86]. Indeed, two such genes, *PTCH1* and *HHIP,* are transcriptionally repressed by DNMT1 [87] or the UHRF1/DNMT1 complex [147], respectively.

### 2.3. Transcription Factors

In general, gene expression is regulated by the cooperative binding of multiple transcription factors (TFs). This enables precise gene expression regulation and cooperative gene activation [148]. In this section, we will discuss those TFs that interact and cooperate with GLI proteins in gene transcription. Though TFs are generally difficult to drug [10], many of these GLI-interacting TFs, such as AP-1 and MKL, have already been targeted by small molecular inhibitors.

#### 2.3.1. Serum Response Factor-Megakaryoblastic Leukemia 1 Complex

Serum response factor (SRF) was identified as a GLI co-activator, based on its enriched chromatin occupancy at GLI-target genes and its selective overexpression in SMO-inhibitor-resistant BCC [58]. In BCC, both SRF and its co-activator Megakaryoblastic leukemia 1 (MKL1) physically interact with GLI1 and co-occupy the regulatory regions of a subset of GLI-target genes [58]. Furthermore, inhibition of SRF or MKL1 by knockdown or treatment with the MKL inhibitor CCG-1423 attenuates the growth of SMO inhibitor resistant BCC cells ([58] and Table 2). Importantly, another MKL1 inhibitor, CCG-203971, also represses GLI signaling and BCC growth in vivo ([58] and Table 2).

#### 2.3.2. Activator Protein 1 Complex

Activator Protein 1 (AP-1) is a heterodimeric transcription factor complex composed of proteins belonging to the JUN (JUN, JUNB, and JUND) and FOS families (FOS, FOSB, FOSL1, and FOSL2) (reviewed in [149,150]). Among these subunits, JUN interacts with GLI2 but not GLI1 to enhance the expression of a subset of GLI-target genes, including *JUN* itself [92]. This GLI2-JUN interaction depends on JUN phosphorylation by JNK, which can be disrupted by the JNK inhibitor SP600125 [92]. In SMO-inhibitor-resistant BCC, the mRNA expression of *JUN*, *JUNB*, *JUND,* and *FOSL2* is enriched [93]. Consistent with AP-1 potentiating GLI activity, knockdown of *JUN*, *JUNB*, *JUND*, and *FOSL2* levels or treatment with AP-1 inhibitors (T5224 and SR11302) decreases *GLI1* expression and viability in BCC cells ([93] and Table 2).

#### 2.3.3. SMAD Complex

SMADs are transcription factors in the TGF-β signaling pathway, where they function as heterotrimeric complexes comprised of SMAD4 and two other members of the SMAD family (reviewed in [151]). The SMAD complex also directly associates with GLI proteins to modulate their transcriptional activity. Specifically, SMAD2 and 4 were shown to physically interact with GLI1 along with PCAF in pancreatic cancer cells, and enhance GLI1-dependent *BCL2* transcription through PCAF-mediated epigenetic activation [94]. In contrast, SMAD5 can also negatively regulate the HH signaling pathway, as its overexpression reduces the SHH-stimulated proliferation of cerebellar granular cell progenitors [95]. The repressor form of GLI3 also associates with SMAD1-4, albeit in a poorly understood way [96]. How different SMAD complexes regulate GLI transcriptional activity, and especially if different compositions of such complexes possess distinct regulatory effects on GLI activity, remains undetermined.

#### 2.3.4. GATA Binding Protein-Friend of GATA Complex

Six GATA binding proteins (GATA1-6) have been identified, which fall into two subgroups: GATA1/2/3 and GATA4/5/6 [152]. While GATA1/2/3 function in hematopoietic stem cells, GATA4/5/6 function in mesoderm and endoderm-derived tissues [152]. GATA proteins are assembled into transcriptional complexes with multiple other components, including Friend of GATA (FOGs), to enhance or repress transcription [153]. All three members of the GATA4/5/6 subgroup repress GLI-mediated transcription. Specifically, GATA6 localizes on regulatory regions of GLI-target genes and cooperates with FOG2 (also known as ZFPM2) to repress their expression [97]. Consistent with GATA6 negatively regulating GLI activity, GATA6 physically interacts with the repressor form of GLI3 to facilitate its nuclear localization and repressor activity [98]. In addition to GATA6, GATA4 can interact with GLI1 and 3, as shown by a mammalian two-hybrid assay [99]. Further, GATA4 and 5 co-occupy the proximal GATA- or GLI-binding sites of the *GLI1* locus with FOG1 (also known as ZFPM1), and *FOG1* knockdown relieved this inhibition of *GLI1* expression [99]. Thus, just as FOG2 cooperates with GATA6, FOG1 cooperates with GATA4/5 to repress GLI-target genes.

#### 2.3.5. SUFU-Containing Complexes

While SUFU is not a transcription factor, we have included it in this section because of its ability to repress GLI transcriptional activity in the nucleus. SUFU associates with all three GLIs [100,101] to regulate the activity of GLI proteins at multiple levels (see above and [106,154,155]), including via its ability to inhibit GLI transcriptional activity [102,103,104]. It interacts with GLI1 and GLI3 on DNA to enhance their DNA binding affinities [103,105]. Thus, SUFU both inhibits GLI1-mediated transcriptional activation and enhances GLI3-mediated transcriptional repression [102]. SUFU’s function as a transcriptional co-repressor of GLIs is likely modulated by multiple other components within the GLI-SUFU complex, consistent with a recent model in which SUFU acts as a chaperone for GLI function [103,156]. Specifically, SIN3A and SAP18 are present in the GLI-SUFU complex, which potentially recruits HDACs to achieve the transcriptional inhibition on GLI-targeted genes (discussed above and [102,103]). Similarly, GATA zinc finger domain containing 2B (GATAD2B or P66β) and HDAC1 have been found to associate with SUFU, which can repress GLI transcriptional activity [104]. Furthermore, SUFU also positions the co-activator MYC binding protein (MYCBP) onto GLI-target genes [104], suggesting a more complicated role for SUFU in GLI-mediated transcription.

#### 2.3.6. Potential GLI Complexes

Besides the well-established multi-protein GLI transcriptional complexes discussed thus far, two additional GLI transcriptional co-factors (Atonal bHLH transcription factor 1 (ATOH1) and Nanog homeobox (NANOG)) have been described. *ATOH1* is overexpressed in SHH-MB, where it co-occupies similar genomic loci as GLI2 [106]. Consistent with ATOH1 acting as a GLI2 co-activator, it can enhance the effects of activated GLI signaling to promote of MB growth [107]. NANOG interacts with GLI1 and the repressor form of GLI3 [108]. Further, *NANOG* knockdown can decrease *GLI1* expression [109], suggesting that NANOG positively regulates GLI signaling. However, this regulation likely occurs in a context dependent manner as NANOG has been also shown to represses GLI activity [108].

## 3. Conclusions

**Figure 4:**
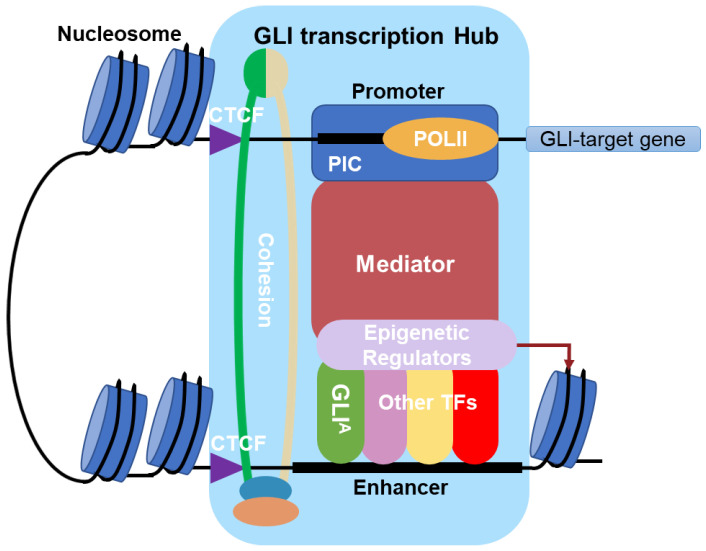
GLI transcription hub/condensate model. The activator forms of GLI (GLI^A^) dynamically interact with transcription co-activators in a local hub to mediate target-gene transcription.

In this review, we have focused on the regulation of GLI-mediated transcription, the final step in the HH signaling pathway. We have described a series of multi-protein transcriptional complexes capable of activating or repressing GLI transcriptional activity in a context dependent fashion. Although we categorized GLI transcriptional cofactors into distinct complexes, recent models of transcriptional regulation have suggested that a high local concentration of transcription complexes, including the general transcription machinery, epigenetic regulators, and TFs, may all be present in dynamic condensates or hubs (reviewed in [157]). Thus, it is likely that these distinct multi-protein complexes also interact with each other at specific times during transcription (Figure 4). In summary, this review has summarized the current knowledge regarding how GLI proteins function in multi-protein transcriptional complexes, and has highlighted druggable proteins within these complexes that could be targeted to inhibit GLI activity (Table 2).

## Figures and Tables

**Table 1:** GLI transcriptional cofactors.

Category	GLI Transcriptional Co-Factor/Muti-Protein Complex	Role in GLI-Mediated Transcription	References
General transcription machinery	TFIID	activator	[59,60]
	Mediator	dual-regulator	[61,62,63]
	Cohesin-CTCF	activator	[64,65]
Epigenetic regulators	BRD4	activator	[66,67,68,69,70,71]
	HATs	activator	[72,73]
	HDACs	repressor	[74,75,76]
	PRMT5	repressor	[77,78]
	SETD7	repressor	[79]
	PRC2	repressor	[80,81]
	SWI/SNF	dual-regulator	[80,82,83,84,85]
	UHRF1-DNMT1	repressor	[86,87]
Transcription factors	TP53	repressor	[60]
	SOX2	activator	[71,88,89,90,91]
	SRF-MKL1	activator	[58]
	AP-1	activator	[92,93]
	SMAD1-5	dual-regulator	[94,95,96]
	GATA4-6	repressor	[97,98,99]
	SUFU	repressor	[100,101,102,103,104,105]
	ATOH1	activator	[106,107]
	NANOG	dual-regulator	[108,109]

**Table 2:** GLI transcriptional complex inhibitors.

Target	Inhibitor	Preclinical Study	Clinical Study
TAF9	FN1-8	lung cancer [59]	n.a.
BRDs	JQ1	MB, BCC, ATRT [69], melanoma [68]	n.a.
	I-BET151	MB [67]	NCT02630251 (terminated): advanced or recurrent solid tumors
	CPI203	PDAC [66]	n.a.
	BMS-986158	MB [71]	NCT03936465: Solid tumor, childhood; lymphoma; brain tumor, pediatric
			NCT04817007: myelofibrosis
			NCT02419417: advanced tumors
			NCT05372354: multiple myeloma
	MZ1	melanoma [68]	n.a.
	ZEN-3365	AML [118]	n.a.
DNMT1	5-Azacytidine	MB [86,87]	FDA-approved for MDS, AML, JMML
	5-aza-2′-deoxycytidine	MB [119]	FDA-approved for MDS
MKL1	CCG-1423	BCC [58]	n.a.
	CCG-203971	BCC [58]	n.a.
AP-1	T5224	BCC [93]	n.a.
	SR11302	BCC [93]	n.a.

Only inhibitors that have been confirmed to attenuate GLI activity are listed. Data was collected from www.clinicaltrials.com accessed on 6 October 2022. Abbreviations: MB, medulloblastoma; BCC, basal cell sarcoma; ATRT, atypical teratoid/rhabdoid tumor; PDAC, pancreatic ductal adenocarcinoma; AML, acute myeloid leukemia; MDS, Myelodysplastic syndromes; JMML, juvenile myelomonocytic leukemia; n.a., not available.

## Data Availability

Not applicable.
